# Direct structure determination of vemurafenib polymorphism from compact spherulites using 3D electron diffraction

**DOI:** 10.1038/s42004-022-00804-2

**Published:** 2023-01-23

**Authors:** Shuting Li, Molly Lightowler, Xiao Ou, Siyong Huang, Yifan Jiang, Xizhen Li, Xiaodong Zou, Hongyi Xu, Ming Lu

**Affiliations:** 1 School of Pharmaceutical Sciences, Sun Yat-sen University, Guangzhou, China; 2 Department of Materials and Environmental Chemistry, Stockholm University, Stockholm, Sweden

**Keywords:** Crystal engineering, Drug discovery and development, X-ray diffraction

## Abstract

The spherulitic morphology is considered to be the most common morphology of crystalline materials and is particularly apparent in melt-crystallized products. Yet, historically, the polycrystalline nature of spherulites has hindered successful crystal structure determination. Here, we report the direct structure determination of a clinical drug, vemurafenib (VMN), in compact spherulite form using 3D electron diffraction (3D ED). VMN has four known polymorphs. We first solved the crystal structures of α-, β-, and γ-VMN from compact spherulites using 3D ED, and the resulting structures were highly consistent with those obtained by single-crystal X-ray diffraction. We then determined the crystal structure of δ-VMN—the least stable polymorph which cannot be cultivated as a single crystal—directly from the compact spherulite sample. We unexpectedly discovered a new polymorph during our studies, denoted as ε-VMN. Single crystals of ε-VMN are extremely thin and not suitable for study by X-ray diffraction. Again, we determined the structure of ε-VMN in a compact spherulite form. This successful structure elucidation of all five VMN polymorphs demonstrates the possibility of directly determining structures from melt-grown compact spherulite samples. Thereby, this discovery will improve the efficiency and broaden the scope of polymorphism research, especially within the field of melt crystallization.

## Abstract (web summary)

The spherulite morphology is common among crystalline materials and melt-crystallized products, however, the structural determination of spherulites by X-ray diffraction remains difficult due to their polycrystalline nature. Here, the authors report the direct structure determination of the polymorphism of vemurafenib in compact spherulite form using 3D electron diffraction.

## Introduction

Spherulites are defined as radially polycrystalline aggregates with an outer spherical envelope^1^. Spherulite formation begins from a single crystal nucleus that grows spherically outwards through the non-crystallographic branching of individual fibrils^2–4^ and can occur from melts^5^, solids^6^, solutions^7^, and gels^8^. Almost every crystalline substance can exhibit spherulitic growth, including elements^9^, minerals^10^, inorganic crystals^11^, small molecule organic compounds^12^, polymers^13^, and proteins^14,15^. Crystal deposits in the human body responsible for medical conditions such as kidney stones^16^, arthrosis (osteoarthritis)^17^, bladder stones^18^, and brain sections from patients with Creutzfeldt-Jakob disease^19^ also share spherulitic morphologies. The polycrystalline nature of spherulites has hindered structure determination from spherulite samples. In some cases, the term *spherulites* has been used pejoratively to describe a failed attempt to prepare a single crystal^20^—indicating that spherulite formation is often considered to be a nuisance during structural studies.

Melt crystallization has shown great potential for application in pharmaceutical polymorph screening^21–34^. By suppressing nucleation whilst providing a large driving force, melt crystallization can access regions of the potential energy landscape inaccessible by traditional methods^35^. For example, nicotinamide, a naturally occurring form of vitamin B3, yields nine polymorphs from melts^21^, whereas only two polymorphs can crystallize from solution^36^. For deltamethrin, the most commonly used insecticide for malaria control, simply melting and cooling the commercial materials yielded a metastable polymorph which exhibits ten times more efficacy against the Anopheles mosquito than the commercial polymorph^30^. The majority of melt-crystallized polymorphs grow as compact spherulites^3,25–28,31^ in a wide temperature range below *T*_max_ (a temperature at which crystal growth rate reaches maximum, usually 0.94 of melting temperature)^1,37,38^, which makes it challenging to cultivate single crystals and, in turn, determine their crystal structures.

There are only a few examples of small molecule crystal structures solved from spherulite samples^25,26,29^. Achieving direct structure solution from powder X-ray diffraction (PXRD) is difficult because the three-dimensional diffraction data are compressed into one dimension, which causes peak overlap and prevents the accurate extraction of individual reflection intensities^39^. Instead, to be successfully solved, those structures required high-quality synchrotron PXRD data combined with crystal structure prediction (CSP) methods. Other attempts to solve crystal structures from spherulite samples using synchrotron PXRD data and CSP methods gave inconclusive unit cell parameters^23,28^, unit cell parameters that could not be matched to any forms predicted in CSP searches^25^ or showed contamination from other polymorphs^25^. These examples show that even after securing the use of synchrotron facilities and spending large amounts of time applying CSP methods, the results gained can be inconclusive.

Recently, we established a general strategy for cultivating single crystals from melt microdroplets that involves growing the crystal at a high temperature (0.97–0.99 of the melting temperature)^40^. Despite a high success rate in our lab^21,32,34,41^ and other groups^22,24,42,43^, this method is incompatible with metastable polymorphs that undergo a solid-solid phase transition when heated.

**Fig. 1:**
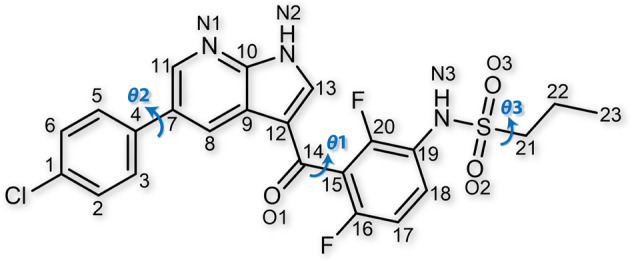
Chemical structure of vemurafenib. Three arrows represent the torsion angles of *θ*1 (C12-C14-C15-C20), *θ*2 (C3-C4-C7-C8), and *θ*3 (N3-S-C21-C22), respectively.

Vemurafenib is a small molecule competitive inhibitor of BRAF, a protein that stimulates cell division (Fig. 1)^44^. In 2011, it was the first drug to gain regulatory approval in the U.S. for the specific treatment of BRAF-mutated melanomas (approximately 60% of human skin melanomas)^45^. The commercially available raw material is the most stable polymorph, α-VMN. In 2016, we discovered three metastable polymorphs (β, γ, and δ) from melts^33^. The spherulitic nature of these polymorphs hindered structure determination until recently. After employing our melt microdroplet strategy, we successfully cultivated single crystals of α-, β- and γ-VMN with sufficient size and quality for structure determination by single-crystal X-ray diffraction (SCXRD) (Supplementary Fig. 1a–c). However, we struggled to obtain a single crystal of δ-VMN as it always transformed to the more stable γ-VMN upon heating and could therefore only be cultivated in spherulite form (Supplementary Fig. 2).

3D electron diffraction (3D ED), also referred to as microcrystal electron diffraction (MicroED), is a powerful method for structure determination of small molecules^34,46–57^. 3D ED has several unique advantages that are particularly relevant to polymorphism research. Firstly, owing to the strong interaction of electrons with matter, very small crystals (micrometres or less) in microgram quantities can be studied^58^. Secondly, the ability to operate the microscope in both imaging and diffraction modes allows individual crystals to be selectively studied, which is ideal for polymorphic mixtures with crystals displaying varying morphologies^52^. Finally, both sample preparation and data acquisition are fast, enabling many crystals to be studied quickly and facilitating the study of beam-sensitive samples^34,46,49–51,53–55,59^, metastable samples^53,57^ and in situ crystallization experiments^52,56^. Often, 3D ED data are collected on dispersed microcrystals, and previously 3D ED has been employed to solve crystal structures from urchin-like proteins^60,61^ and hedgehog-like zeolites^62^ (open spherulites with radii (*L*_s_) to fibril thickness (*h*) ratios of 10^2^–10^3^)^1^. However, historically it has been challenging to determine crystal structures from compact spherulites due to the fine fibrils with a very high *L*_s_/*h* ratio of 10^3^–10^5^ ^1^.

The failure to grow suitable single crystals of δ-VMN led us to explore if 3D ED can directly determine the structure of δ-VMN from compact spherulites, the most common morphology of melt crystallization products. In this work, we began by studying α-, β- and γ-VMN, as the structures obtained by 3D ED could be compared to those obtained by SCXRD. We then applied the method to δ-VMN, which can only be cultivated as a compact spherulite, and a new ε-VMN, which was unexpectedly discovered during the course of this work, to demonstrate further the advantages of sidestepping the bottleneck step of single crystal growth. This successful structure elucidation of all five VMN polymorphs demonstrates the possibility of determining crystal structure from spherulite samples directly and it will improve efficiency and broaden the scope of crystallographic research in both academia and industry, where spherulites are common products.

## Results

### X-ray crystallography and the discovery of ε-VMN

Single crystals of α-, β-, and γ-VMN were successfully cultivated by the microdroplet melt crystallization technique. SCXRD data were collected at 298 K (α- and β- VMN) and 250 K (γ-VMN), and the structures were solved using direct methods. ε-VMN The crystallographic and refinement data can be found in Table 1 and Supplementary Data 1.

**Table 1:** Experimental crystallographic and refinement data of the five VMN polymorph crystal structures solved in this work^[a]^.

	α-VMN		β-VMN		γ-VMN		δ-VMN	ε-VMN
Discovery, year^[b]^	SC, 2015^84^		MC, 2016^33^		MC, 2016^33^		MC, 2016^33^	MC, this work
Structure	SCXRD,	3D ED,	SCXRD,	3D ED,	SCXRD,	3D ED,	3D ED,	3D ED,
elucidation, year^[c]^	This work	This work	This work	This work	This work	This work	This work	This work
Sample type^[d]^	SC	CS	SC	CS	SC	CS	CS	CS
CCDC No.	1893045	2169341	1893062	2169342	1893054	2169343	2169349	2169344
Temperature (K)	298	293	298	293	250	293	293	293
Crystal system	Monoclinic		Monoclinic		Triclinic		Monoclinic	Monoclinic
Space group	*P*2_1_/*n* (14)		*P*2_1_/*c* (14)		*P*$\bar{1}$ (2)		*P*2_1_/*c* (14)	*P*2_1_ (4)
Z’, Z	1, 4		2, 8		1, 2		1, 4	2, 4
*a* (Å)	19.1792(2)		9.9495(1)		7.6759(1)		14.0790(1)	4.885(1)
*b* (Å)	5.6702(1)		13.0705(1)		10.3177(1)		8.5200(1)	22.377(1)
*c* (Å)	20.1250(2)		35.8432(3)		14.9190(2)		21.5220(1)	20.428 (1)
*α* (°)	90		90		98.481(1)		90	90
*β* (°)	96.096(1)		94.439(1)		100.478(1)		109.931(1)	94.952(2)
*γ* (°)	90		90		105.475(1)		90	90
Volume (Å^3^)	2176.22		4647.24		1095.51		2427.00	2224.70
Density (g·cm^−3^)	1.495		1.400		1.485		1.341	1.463
Total reflections	12783	10182	47896	9875	20854	10606	565 (653)	4912
Unique reflections	4284	2361	9370	3840	4419	3284	288 (230)	2783
Resolution (Å)	0.79	0.90	0.79	1.00	0.79	0.80	1.4 (2.0)	1.05
Completeness (%)	99.8	75.4	99.8	79.1	99.9	72.6	34.4 (71.0)	73.9
*R*_int_	0.0337	0.1788	0.0285	0.1305	0.0298	0.2033	0.1160 (0.1478)	0.186
I/sigma	26	4.68	42.4	3.78	50.8	3.31	3.66 (8.40)	5.30
CC1/2^[e]^	NA	98.2	NA	98.7	NA	96.9	98.0 (98.5)	97.8
No. of parameters	299	299	597	507	299	299	97	268
No. of restraints	0	12	3	226	0	48	97	303
*R*_1_ (all)	0.0475	0.2321	0.0683	0.2720	0.0428	0.2540	0.3339	0.2754
wR_2_	0.1247	0.4200	0.1829	0.4816	0.1100	0.4277	0.6269	0.4745
No. of datasets	1	3	1	2	1	6	1 (4)	3
RMSD_15_ (Å) ^[f]^	0.091	0.117	0.17	0.208	0.113	0.164	0.418	0.271

^[a]^The unit cell parameters of α-, β- and γ-VMN are taken from the SCXRD data, whilst those of δ- and ε-VMN were refined against the PXRD data. The values for δ-VMN are the data used for the structure refinement (1.4 Å), whilst the values in parentheses are the data used during simulated annealing for the structure solution (2 Å).

^[b]^Year and method of discovery: SC, solution crystallization; MC, melt crystallization.

^[c]^Method used for structure elucidation: SCXRD, single-crystal X-ray diffraction; 3D ED, 3D electron diffraction.

^[d]^Sample type used for structure elucidation: SC, single crystal; CS, compact spherulite.

^[e]^NA, not available.

^[f]^RMSD15 values are calculated between the experimental structure and the density functional theory (DFT) energy minimized structures using Mercury software.

**Fig. 2:**
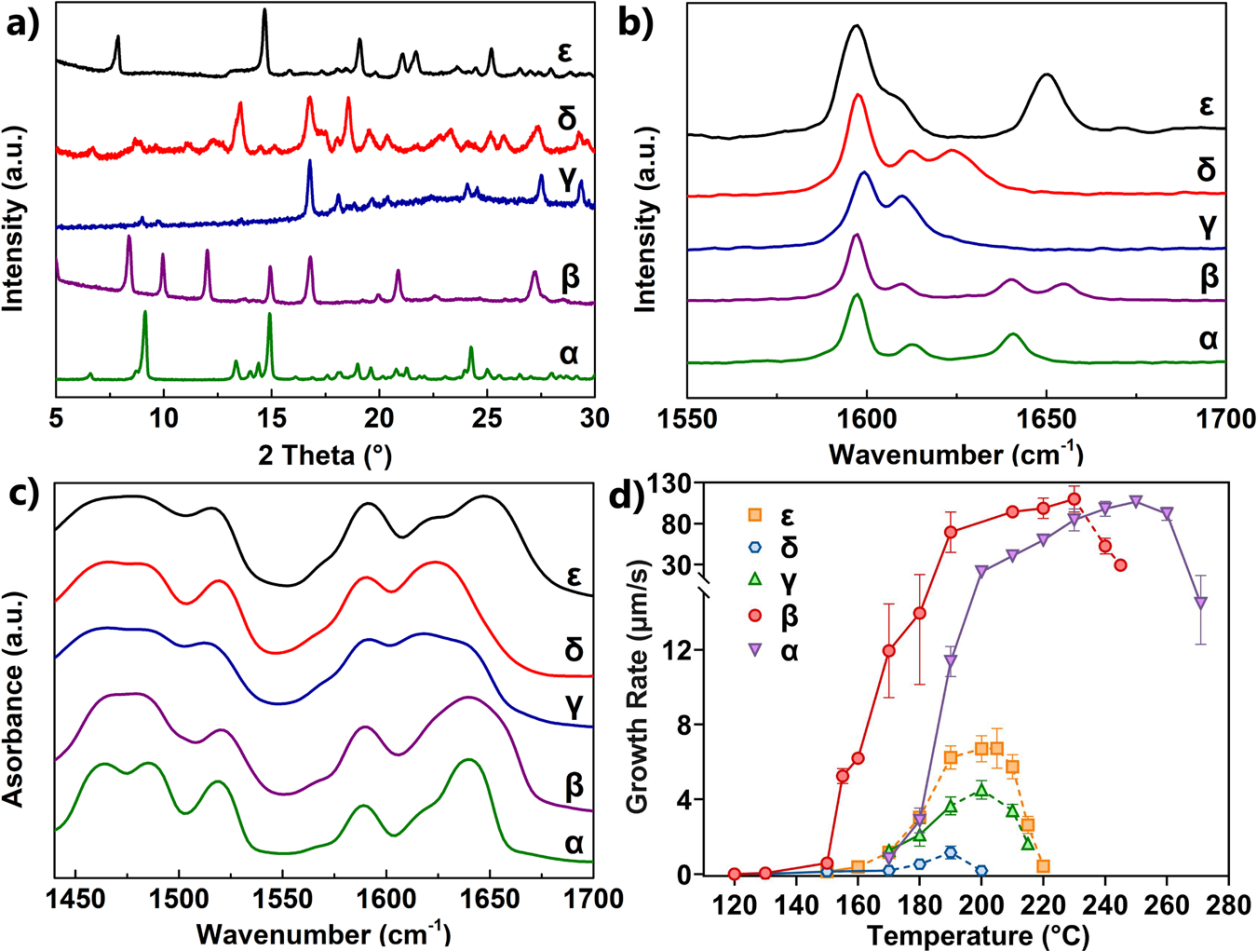
Characterization of the five VMN polymorphs. **a** PXRD patterns. **b** Raman spectra. **c** FTIR spectra. **d** Growth rates (the dotted line indicates the temperature region where the polymorphs cannot spontaneously nucleate; each experiment has been repeated at least three times, and the data obtained were consistent).

δ-VMN is the least stable polymorph. Attempts to grow a single crystal of δ-VMN from the melt droplet always resulted in a single crystal of γ-VMN. The combination of Raman microscopy with a hot stage revealed that δ-VMN converts to γ-VMN when heated beyond 200 °C (Supplementary Fig. 2). During the preparation of δ-VMN, we observed that a spherulite spontaneously nucleated at 150 °C with a very low nucleation density and a unique melting point of 227 °C (α: 272 °C; β: 254 °C; γ: 225 °C). Further characterization also revealed a unique PXRD pattern, Raman and Fourier-transform infrared (FTIR) spectra, and a different growth rate (Fig. 2 and Supplementary Fig. 4)—all of which indicated the discovery of a new polymorph, which we named ε-VMN. The single crystal of ε-VMN was difficult to cultivate since the optimal growth temperature is 215 °C, and at this temperature, α- and β-VMN often nucleate spontaneously. Besides that, the needle-like single crystal of ε-VMN is less than 10 μm wide (Supplementary Fig. 1d) and was not suitable for study by SCXRD.

### Structure elucidation from spherulites by 3D ED

**Fig. 3:**
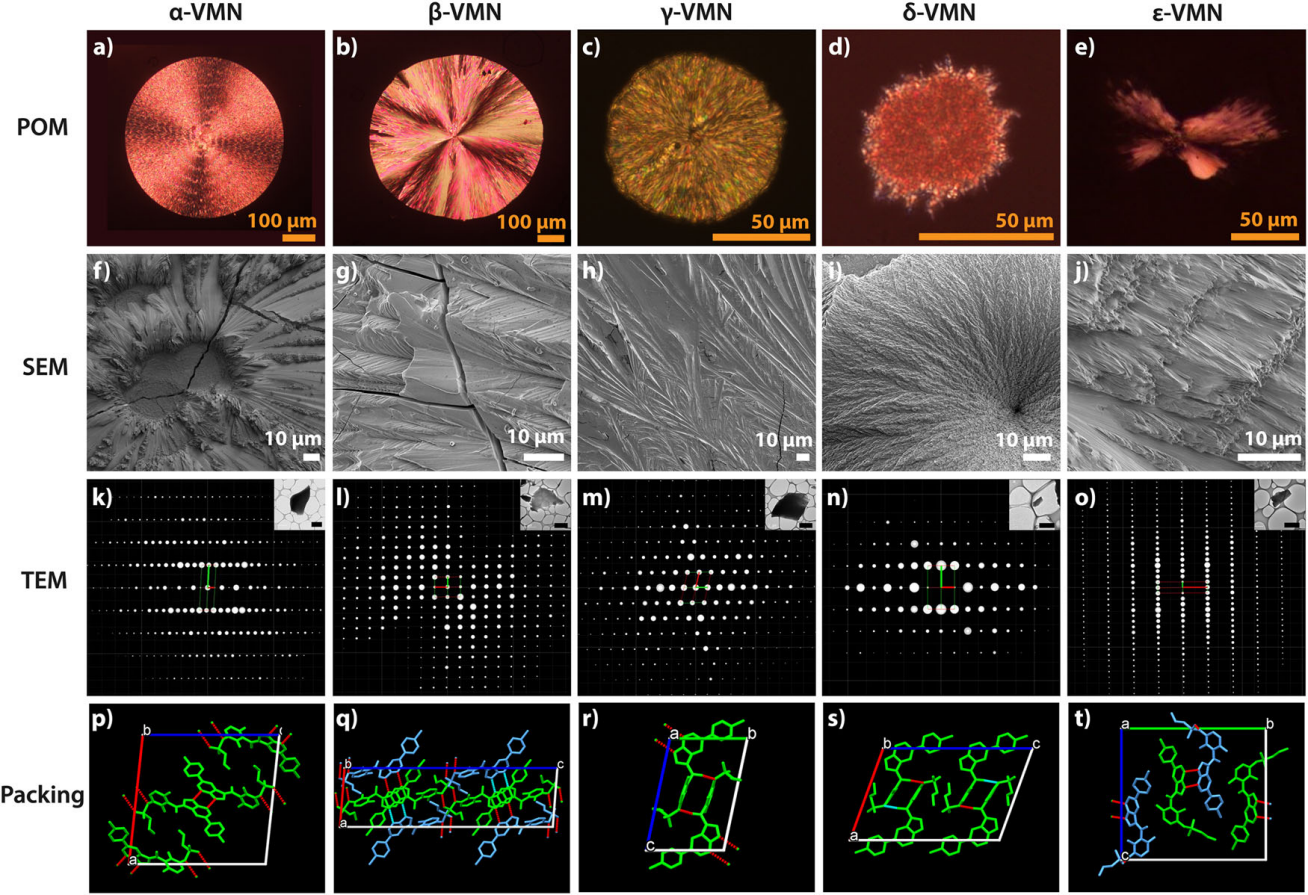
Direct structure determination of the five VMN polymorphs using 3D ED from compact spherulite samples. From left to right: α-, β-, γ-, δ-, and ε-VMN. **a**–**e** POM images. **f**–**j** SEM micrographs. **k**–**o** 3D reconstructed reciprocal lattice (viewed along the c*-axis) with transmission electron microscopy (TEM) micrographs of the fragments of compact spherulites for data collection (scale bar is 2 μm). **p**–**t** Crystal packing. Colours in **q** and **t** (light blue and green) are used to differentiate crystallographically independent molecules. Diffuse streaks along b*-axis in **o** caused by stacking disorder are removed for clarity.

To verify whether crystal structures from melt-grown spherulite samples can be solved using 3D ED, we began by studying polymorphs α-, β- and γ-VMN. We prepared compact spherulite samples on coverslips using a hot-stage microscope, as shown in Fig. 3. Polarized optical microscopy (POM) and scanning electron microscopy (SEM) were employed to characterize the morphologies of these compact spherulite samples (Fig. 3a–j and Supplementary Fig. 5).

After sample preparation, 3D ED data were collected on isolated fragments of varying morphologies and sizes (Fig. 3k–o and Supplementary Fig. 6). Since the structures of α-, β- and γ-VMN had been solved previously, the unit cell parameters and space group symmetries could be taken directly from the SCXRD results during data processing (Supplementary Fig. 7). After merging data from multiple crystals, the structures were solved using SHELXT^63^ and refined using SHELXL^64^ through the ShelXle interface. The experimental crystallographic and refinement data can be found in Table 1 and Supplementary Data 1. The structure models of α-, β-, and γ-VMN obtained using SCXRD gave a higher resolution, higher completeness, and lower R-factor than those obtained by 3D ED. The differences in the crystallographic data between the two methods have been well-documented previously and owe to the fundamental differences between the radiation types and experimental techniques^65^. Dynamical refinement has been shown to improve results, including better agreement factors, higher accuracy of atomic positions, and decreased noise in the difference electrostatic potential maps, however, it was not applied in this study^66–68^. Nonetheless, the structure models solved by the two methods are very similar with minimal values of a root mean square deviation of the 15 overlaying molecules (RMSD_15_) (0.062 Å for α-VMN, 0.165 Å for β-VMN and 0.090 Å for γ-VMN). The largest differences between the structures were amongst the terminal and flexible alkyl chains (Supplementary Fig. 8). The PXRD patterns simulated from the structures solved by 3D ED show differences in peak intensities when compared to the experimental PXRD patterns (Supplementary Fig. 3). These differences are likely due to the anisotropic growth and preferred orientation of the individual fibrils of the spherulites. Despite this, the simulated PXRD patterns from the models solved by SCXRD and 3D ED are similar. From these results, we can confirm that the structures obtained from compact spherulites by 3D ED are consistent with those obtained from single crystals by SCXRD, demonstrating direct crystal structure determination of a small organic molecule in melt-grown spherulite form.

After verifying the method, we moved on to test its advantages for structure determination of polymorphs that can only be obtained in compact spherulite form and whose structures, therefore, remain unknown. Differing from the α-, β-, and γ-VMN polymorphs, spherulites of δ-VMN have a unique morphology. SEM micrographs show that the irregularly-shaped δ-VMN spherulites with sunken centres have a rough surface (Fig. 3i and Supplementary Fig. 5g, h). Additionally, the δ-VMN spherulites did not display the typical ‘Maltese cross’ pattern of light extinction under POM (Fig. 3d).

Crushed crystals of δ-VMN had a width typically less than 0.8 μm after sample preparation. Along with the differing spherulite morphology, δ-VMN crystals are less ordered than the previously solved polymorphs, as shown by the lower resolution of 3D ED data (~2.0 Å) and broadened peaks in the PXRD pattern (Supplementary Fig. 3). Despite this, the reflections in the 3D ED data could be indexed, giving a monoclinic unit cell and space group *P*2_1_/c (No. 14) (Supplementary Fig. 7). With such low resolution, the data were unsuitable for structure solution by direct methods. For low resolution data, simulated annealing is a good alternative method when the molecule connectivity is known. After merging four data sets cut to 2 Å resolution (71.0% completeness), simulated annealing was run as implemented in the programme Sir2014^69^. The resulting initial structure models were all similar and chemically reasonable. The initial unit cell parameters from the 3D ED data were further refined against the PXRD data to give more accurate unit cell parameters of *a* = 14.079 (1) Å, *b* = 8.520 (1) Å, *c* = 21.522 (1) Å, *α* = 90 °, *β* = 109.931 (1) °, *γ* = 90 ° (Supplementary Fig. 9 and Supplementary Table 1). Since our data/parameter ratio was too low to perform a full refinement, we chose to confirm the crystal packing by performing the structure refinement using the only high-resolution dataset (1.4 Å), which converged to a final *R*_1_(all) value of 33.4% (Table 1 and Supplementary Data 1). There is a discrepancy between the experimental PXRD pattern and that simulated from the model (Supplementary Fig. 3). The peaks in the experimental pattern are much broader due to the small size of the individual fibrils.

Compact spherulites of ε-VMN are built from extremely fine fibrils (Fig. 3f–j and Supplementary Fig. 5i, j). Although the microcrystals diffracted to a high resolution (0.95 Å), they were disordered, as indicated by the presence of diffuse streaks in the reconstructed 3D reciprocal space. The diffuse scattering in the 3D ED data occurs along the b*-direction in every second diffraction row with odd *l* values (Supplementary Fig. 12). This diffuse scattering indicates that the disorder is related to stacking faults of the dimers with a shift vector of *c* = ½ between planes perpendicular to the *b*-axis. This disorder led to difficulties in determining the correct unit cell parameters and space group symmetry during data processing, as it was hard to establish where the diffused intensities are centred in reciprocal space. However, after numerous attempts, one combination of unit cell parameters in a monoclinic setting and space group *P*2_1_ (No. 4) (Supplementary Fig. 7) produced a reasonable initial model after structure solution using SHELXT^63^. The unit cell parameters were refined against the PXRD data to give final unit cell parameters of *a* = 4.885 (1) Å, *b* = 22.337 (1) Å, *c* = 20.428 (2) Å, *α* = 90 °, *β* = 94.952 (2) °, *γ* = 90 ° (Supplementary Fig. 10). Merging data from three individual crystals from the compact spherulite resulted in 73.9% completeness. The refinement converged to an R1(all) value of 27.5% (Table 1). In the crystal structure of ε-VMN, doubly hydrogen-bonded 7-azaindole dimers are stacked along the *a*-axis. In addition to the π–π stacking interactions of the azaindole groups, the sulfonamide group of each molecule is hydrogen-bonded to two neighbouring molecules, creating a hydrogen-bonded chain of molecules along the *a*-axis. Between these chains of molecules, no hydrogen bonding occurs, and columns of molecules with only weak interactions are formed (Supplementary Fig. 13). Based on the diffuse scattering and the intermolecular interactions in the crystal structure, we suspect the slippage of columns of molecules along the *c*-axis leads to the diffuse scattering observed in the 3D ED data. The second shift variant of the structure is shown in Supplementary Fig. 13.

Additionally, the calculated PXRD pattern from the final structure model of ε-VMN is somewhat consistent with the experimental PXRD pattern (Supplementary Fig. 3). The missing peaks in the experimental PXRD pattern correspond to the reflections where diffuse streaks occur (*l* = odd values). Therefore, the missing peaks in the PXRD data are also attributed to the diffuse scattering resulting from the disordered structure.

A large number of restraints were applied to both δ- and ε-VMN (Table 1). In each case, the restraints were applied to make the structures more chemically reasonable. The refinement models without applying any restraints are shown in Supplementary Fig. 19 and Supplementary Table 2. The results indicated that the refinements are stable and show similarity to the final models, and thereby, sensible to the diffraction data.

### Optimization of crystal structures by DFT method

**Fig. 4:**
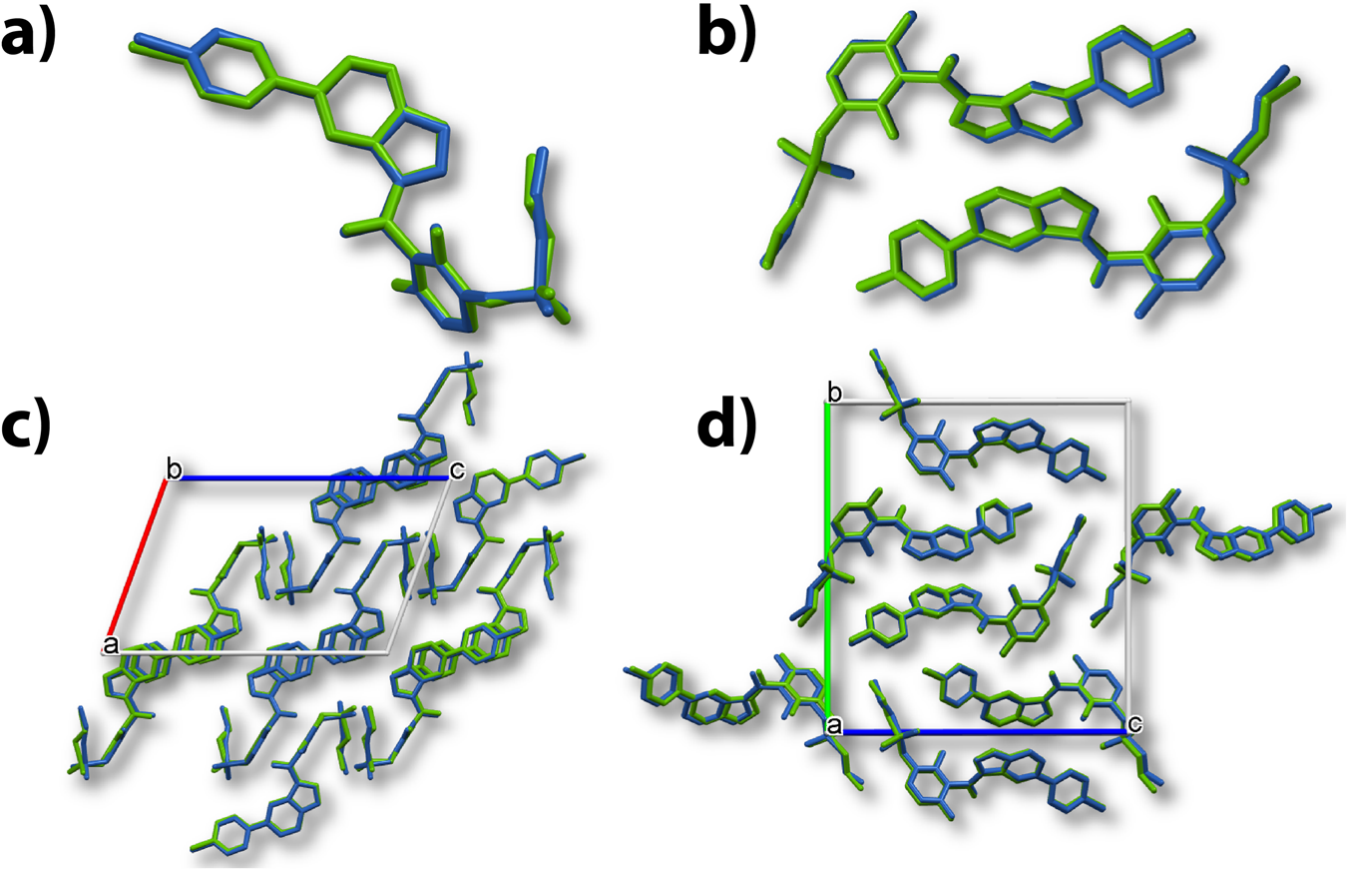
Comparison of structures determined by 3D ED (blue) and energy-minimized structure (green) by DFT optimization. **a** Molecule overlay ofδ-VMN. **b** Molecule overlay of ε-VMN. **c** Crystal packing similarity of δ-VMN. **d** Crystal packing similarity of ε-VMN.

To further confirm the correct structure elucidation for δ- and ε-VMN, both ED structures were energy-minimized by the DFT method, together with the other six structures of α-, β-, and γ-VMN determined by SCXRD and ED (Supplementary Data 2). Molecular overlay and crystal packing similarity between the final DFT optimized structures with the experimental structures were calculated using Mercury software and shown in Fig. 4 and Table 1. Small RMSD_15_ values of all the five polymorphs (0.091–0.418 Å) clearly reflect that the energy-minimized structures match the experimental structures well, and thereby, further confirm the structure validation of five VMN polymorphs.

## Discussion

**Fig. 5:**
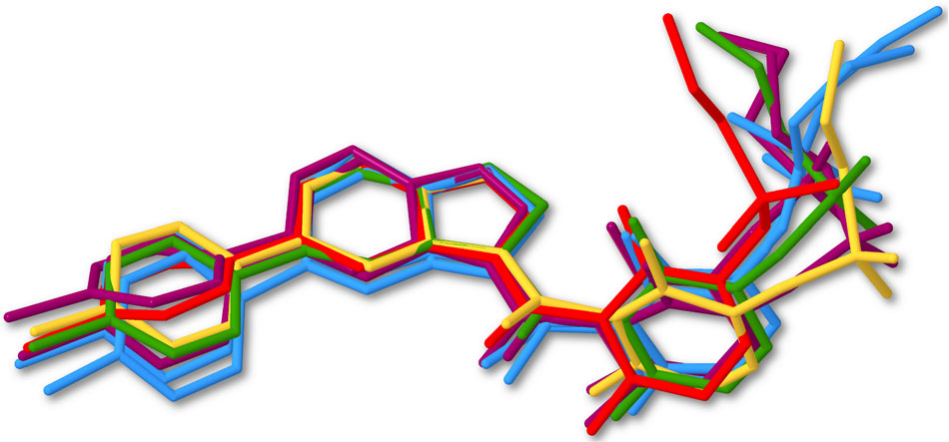
Overlay of the seven conformationally distinct molecules. Red: α-VMN; purple: β-VMN (molecules 1 and 2); yellow: γ-VMN; green: δ-VMN; blue: ε-VMN (molecules 1 and 2), respectively.

The rich polymorphism of VMN results from differences in the molecular conformation of the molecules (Fig. 5). The major conformational difference amongst the VMN polymorphs is caused by the free rotation of the C14-C15 single bond between the carbonyl and difluorophenyl moieties, which can be described by torsion angle *θ*1 (C12-C14-C15-C20) (Fig. 1 and Supplementary Table 3).

Crystal structures of five VMN polymorphs are shown in Fig. 3p–t and Supplementary Figs. 11, 14–18. A planar, doubly hydrogen-bonded 7-azaindole dimer (N2-H···N1) forms between two neighbouring molecules in all VMN polymorphs except for β-VMN, for which the 7-azaindole dimer is nonplanar with torsion angles of 7.53 ° (N2’-N2-N1-N1’) and 7.35 ° (N1-N1’-N2’-N2) (Supplementary Fig. 15). In α- and β-VMN, an additional planar, doubly hydrogen-bonded sulfonamide dimer (S=O3···H-N3) forms between two neighbouring molecules (Supplementary Figs. 14, 15). Although α- and β-VMN have similar hydrogen-bonding networks, the molecular packing is denser in α-VMN (1.495 g·cm^−3^) than in β-VMN (1.400 g·cm^−3^), which may be an important reason for the increased thermodynamic stability of α-VMN^33^. In γ-VMN, an intermolecular fourteen-membered hydrogen-bonded ring forms between the carbonyl and sulfonamide groups (C14=O1···H-N3) (Supplementary Fig. 16), which is less stable than the eight-membered hydrogen-bonded ring formed between the sulfonamide dimers in α- and β-VMN and could be the reason why γ-VMN transforms easily to α- and β-VMN^33^. γ-VMN and δ-VMN have similar bonding networks, although, in δ-VMN, the molecular conformation is unfavourable for ring formation. The distance between the 7-azaindole dimer in δ-VMN is larger than that in γ-VMN, and one of the hydrogen bonds is instead shared with an adjacent molecule (Supplementary Fig. 17), making δ-VMN far less stable than γ-VMN. Once heated above 200 °C, δ-VMN transforms to γ-VMN. We speculate that the C14-C15 single bond rotates upon heating, resulting in a conformational change and the formation of the carbonyl-sulfonamide dimer and thus the phase conversion from δ-VMN to γ-VMN. In ε-VMN, the sulfonamide group is hydrogen-bonded to two neighbouring molecules (S=O2···H-N3), creating a hydrogen-bonded chain of molecules along the *a*-axis (Supplementary Fig. 18). This additional bond may cause the increased stability of ε-VMN overδ-VMN.

Phase transformations between VMN polymorphs were first reported in 2016^33^, but traditional methods could not reveal the mechanisms by which they convert. By successfully determining and analyzing the crystal structures of all five polymorphs, we could elucidate the relationship between crystal structure and phase behaviour in VMN. These results demonstrate that using 3D ED, it is now feasible to bypass the step of single crystal growth and solve crystal structures directly from compact spherulites. It is well known that structure elucidation for polymorphic spherulites discovered from melts has been a difficult and time-consuming process. Considering that there are still several reported polymorphic spherulites^22,70,71^ with unknown crystal structures, we believe that many labs may presently be struggling with the structure elucidation of unreported compact spherulites. This study presents an additional avenue for these researchers. Melt crystallization is a fast, simple, and cheap screening process that can efficiently obtain polymorphs that are difficult to obtain from solution^21–34^, and this work will help to promote the further application of melt crystallization in polymorphism research, for example, in polymorphism screening during drug development. Moreover, for other research fields where compact spherulites are common products, these findings will inspire the use of 3D ED for structure elucidation, thereby promoting crystallographic research across various fields.

## Conclusion

By applying 3D ED along with its ability to achieve structure determination from micrometre-sized crystals, we have directly solved the polymorphic structures of a small molecule organic compound in compact spherulite form. To verify both the applicability and generality of the technique, we showed the minimal differences between the crystal structures of α-, β-, and γ-VMN obtained using 3D ED from compact spherulites to those obtained from single crystals using SCXRD. Following on, we successfully solved the crystal structures of both δ- and ε-VMN—two polymorphs that could not be cultivated as single crystals suitable for study by SCXRD—in spherulite form. By doing so, we demonstrated that the time-consuming and often impossible step of single crystal growth can be bypassed entirely for structure elucidation from melt-grown compact spherulites, which highly facilitates polymorphism research of small organic molecules. Having the ability to compare the crystal structures of all five polymorphs revealed the differences in molecular conformation and packing that lead to the rich polymorphism and gave insights into the phase transformation processes. This powerful and convenient technique will improve efficiency and broaden the scope of crystallographic research in both academia and industry, where spherulites are common products.

## Methods

### Single crystal cultivation

Single crystals of α-, β- and γ-VMN were synthesized by the melt microdroplet method using a Linkam hot stage (THM S600, UK) combined with a Nikon POM (Nikon eclipse lv100N pol, China). The commercial VMN powder, purchased from ChemShuttle (Jiangsu, China), was first melted at 274 °C for a few seconds and then cooled to 265 °C. Once an α-VMN nucleus appeared, the melt droplet was immediately heated to 268 °C to let it grow and consume the melt. For β- and γ-VMN, concomitant seeds of α-, β-, and γ-VMN were prepared by crystallizing VMN supercooled liquid at 170 °C. β- and γ-VMN seeds were then introduced into melt microdroplets at 245 and 215 °C, respectively. The polycrystalline seeds were partially melted at 255 °C (for β-VMN) and 224 °C (for γ-VMN) to yield a single nucleus for each polymorph, which were then harvested at 252 °C (for β-VMN) and 215 °C (for γ-VMN) to yield single crystals. SCXRD data were collected on an XtaLAB Synergy-S diffractometer (Rigaku, Poland) using Cu Kα radiation (λ = 1.54184 Å). Cell refinement and data reduction were carried out by CrysAlisPro 171.40.39^72^. The crystal structures were solved using SHELXT^63^ and refined using SHELXL^64^ through the Olex 2-1.3 interface. The simulated PXRD patterns and RMSD_15_ values were calculated by Mercury software (version 2020.3.0).

### Preparation of spherulite samples

The compact spherulites of α-, β-, and γ-VMN were prepared by seeding crystallization. The raw materials were first fully melted at 280 °C using a Kaisi hot plate and then transferred to a Linkam hot stage equipped with POM at 190 °C. Seeds of α-, β-, and γ-VMN were then introduced to the supercooled liquids and left to consume all the materials. ε-VMN was also prepared by seeding crystallization and was cultivated at 170 °C to form a compact spherulite. It was difficult to obtain pure spherulites of δ-VMN as δ-VMN often transforms to a more stable phase (either α- or β-VMN) *via* a solid-solid phase transition upon exposure to it. To avoid unnecessary nucleation of a more stable phase whilst preparing δ-VMN, we sprinkled the commercial VMN powder on a coverslip and melted the dispersed powder at 274 °C to yield several small melt microdroplets, which we then cooled to 150 °C to allow spontaneous nucleation. At this temperature, α-, β-, and δ-VMN can all potentially nucleate. However, it is possible to yield a crystallographically pure δ-VMN phase if it first nucleated in an isolated melt droplet less than 300 μm in diameter and consumed the melt undisturbed.

### SEM

SEM micrographs were recorded using a JEOL 7000 (JEOL, Japan). To reduce charging effects, the spherulites were first coated with a thin layer of gold using a JEOL JFC-1200 Fine coater (JEOL, Japan) at 10 mA for 60 s prior to data collection and the microscope was operating with a low acceleration voltage of 1 kV.

### 3D ED

TEM grids were first glow discharged for 40 s using a PELCO easiGLOW (Ted Pella, Inc., USA) with a current of 20 mA. Polycrystalline spherulites of VMN polymorphs, prepared by melt crystallization, were crushed between two microscope slides. The TEM grid was gently pressed against the broken fragments multiple times and then lightly tapped to remove the larger fragments. The sample grids were then loaded into the microscope. 3D ED data were collected on a JEOL JEM-2100 LaB_6_ TEM equipped with a fast Timepix hybrid pixel detector (512 × 512 pixels) (Amsterdam Scientific Instruments, Netherlands) operating at 200 kV in selected area electron diffraction (SAED) mode. Data were collected at room temperature using the continuous rotation method^46,59,60,73–75^. The software *Instamatic* was used for electron diffraction data collection^76^. Diffraction data were collected by continuously rotating the crystal at a rate of 1.13° s^−1^. The exposure time was 0.3 s, meaning the individual diffraction images were integrated over 0.34° of reciprocal space. Several data sets collected from different crystals were acquired across a rotation range of −60° to +60 ° and scaled together by XSCALE^77^, in order to improve completeness and I/σ(I) and obtain a single data set suitable for structure solution and refinement. The diffraction patterns were indexed using Rotation ED processing software (REDp)^78^ and integrated and merged using crystallography software (XDS)^77^. The structures of α-, β-, γ-, and ε-VMN were solved using SHELXT^63^. The structure of δ-VMN was solved using the simulated annealing method implemented in the programme Sir2014^69^ using the atom connectivity of γ-VMN as the molecular model together with the merged 3D ED dataset. After 10 runs, the resulting models were all similar and displayed reasonable intermolecular interactions. The initial unit cell parameters of γ- and ε-VMN were refined against PXRD data using the Pawley fit method implemented in the programme TOPAS^79^. All structures were refined using SHELXL^64^ through the ShelXle interface. Since δ-VMN diffracted to such a low resolution (~2 Å), the data/parameter ratio was too low to perform a full refinement. Instead, we confirmed the crystal packing (not fine structural details) in the final structure model by refining the model against a single dataset with the highest resolution (1.4 Å). In this refinement, we used AFIX commands to the lower the number of parameters and showed that the model corresponded well with the *F*(obs) map (Supplementary Fig. 19).

### PXRD

PXRD patterns were recorded using a Rigaku D-MAX/2200 VPC X-ray diffractometer (Rigaku, Japan), using Cu Kα radiation (λ = 1.54184 Å) in 40 kV and 26 mA conditions. The samples were placed on a monocrystalline silicon plate and scanned from 5° to 40° (2θ) with a scanning speed of 6°/min at room temperature.

### Raman spectroscopy

Raman spectra were collected using a DXR3xi Raman confocal microscope (Thermo Scientific, USA) equipped with a 785 nm excitation laser and a high-resolution grating. The laser power was approximately 24.2 mW, with the laser spot diameter around 2 μm. The spectra were recorded from 50 to 1800 cm^−1^ by 500 accumulated scans at a resolution of 2 cm^−1^. All experiments were performed in triplicate.

### FTIR

FTIR spectra were collected in transmission mode using a Nicolet 6700 spectrometer (Thermo Scientific, USA) equipped with a Contiuμm microscope (32× objective). The spectra were accumulated by 64 scans from 600 to 3600 cm^−1^ with a spectral resolution of 4 cm^−1^ and were analyzed using Omnic 8.2.0.387 software. All experiments were performed in triplicate.

### Crystal structure validation and optimization by DFT

Energy minimization with DFT was employed for structure validation. Based on the DFT and the projector augmented-wave (PAW) method^80^ as implemented in the Vienna Ab-initio Simulation Package (VASP), the vdW-DF method^81^ that accounts for the dispersion interactions in molecular crystals^82^ was used to perform the energy minimization with a force convergence criterion of 0.01 eV/Å. A plane-wave basis set^83^ is used with a kinetic energy cutoff of 520 eV and a *k*-point spacing of the Brillouin zone integration of approximately 0.05 Å^−1^. Molecular overlay and crystal packing similarity were calculated using mercury software (version 2020.3.0). RMSD_15_ were employed to quantitively evaluate the packing similarity of the crystal structures and the molecular conformations between the experimental structures and the energy-minimized structures.

## Supplementary information

Supplementary Information

Description of Additional Supplementary Files

Supplementary Data 1

Supplementary Data 2

## Data Availability

The data supporting the findings of this study are included in this article and its Supplementary Information. CCDC deposit numbers 1893045 (α-VMN), 1893062 (β-VMN), 1893054 (γ-VMN), 2169341 (α-VMN), 2169342 (β-VMN), 2169343 (γ-VMN), 2169344 (δ-VMN) and 2169349 (ε-VMN) contain the supplementary crystallographic data for this paper, and the CIFs files are provided as a cif file named Supplementary Data 1. These data are provided free of charge by the Cambridge Crystallographic Data Centre Access Structures service via www.ccdc.cam.ac.uk/data_request/cif. The DFT optimized structures are provided as a cif file named Supplementary data 2.
