# Supplementary material for: Direct structure determination of vemurafenib polymorphism from compact spherulites using 3D electron diffraction
Source: Commun Chem. 2023 Jan 23;6:18. doi: 10.1038/s42004-022-00804-2 (PMC9871043; doi:10.1038/s42004-022-00804-2)
Supplement: Supplementary file 1 — Supplementary Information [file 42004_2022_804_MOESM1_ESM.pdf]

Polymorphism of vemurafenib: Direct structure determination from compact spherulites using 3D electron diffraction

Shuting Li,[a,+] Molly Lightowler,[b,+] Xiao Ou,[a] Siyong Huang,[a] Yifan Jiang,[a] Xizhen Li, [a] Xiaodong Zou,[b] Hongyi Xu,[b*] Ming Lu[a*]

[a] School of Pharmaceutical Sciences, Sun Yat-sen University, Guangzhou, China. E-mail: luming3@mail.sysu.edu.cn
[b] Department of Materials and Environmental Chemistry, Stockholm University, Stockholm, Sweden. E-mail: hongyi.xu@mmk.su.se
[+] These authors contributed equally to this work.
[*] These authors jointly supervised this work.

Electronic Supplementary Information

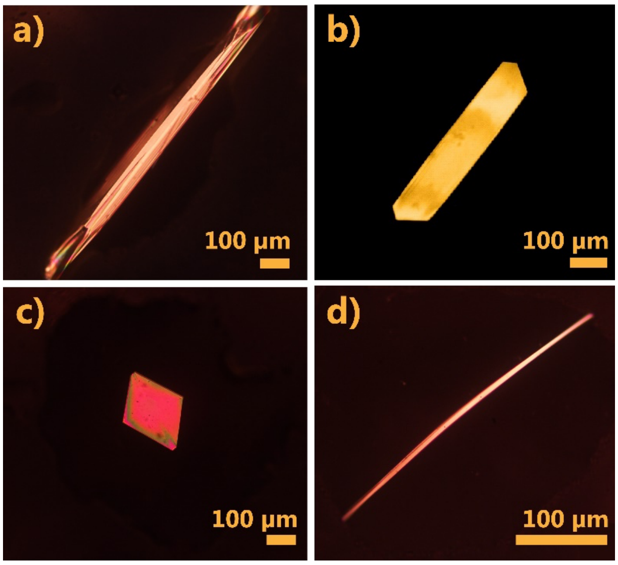

Supplementary Figure 1. POM images of vemurafenib (VMN) single crystals. (a) α-VMN. (b) β-VMN. (c) γ-VMN. (d) ε-VMN.

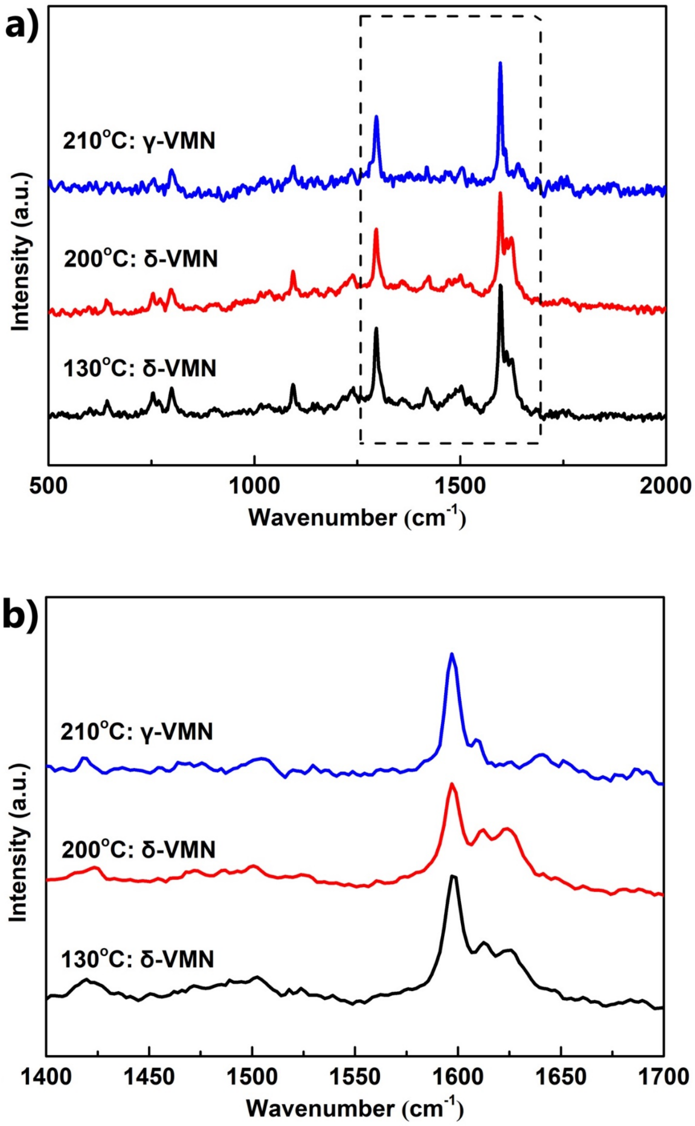

Supplementary Figure 2. Raman spectra showing the δ-to-γ phase transformation of VMN during heating. (a) 500-2000 cm^{-1}. (b) 1400-1700 cm^{-1}.

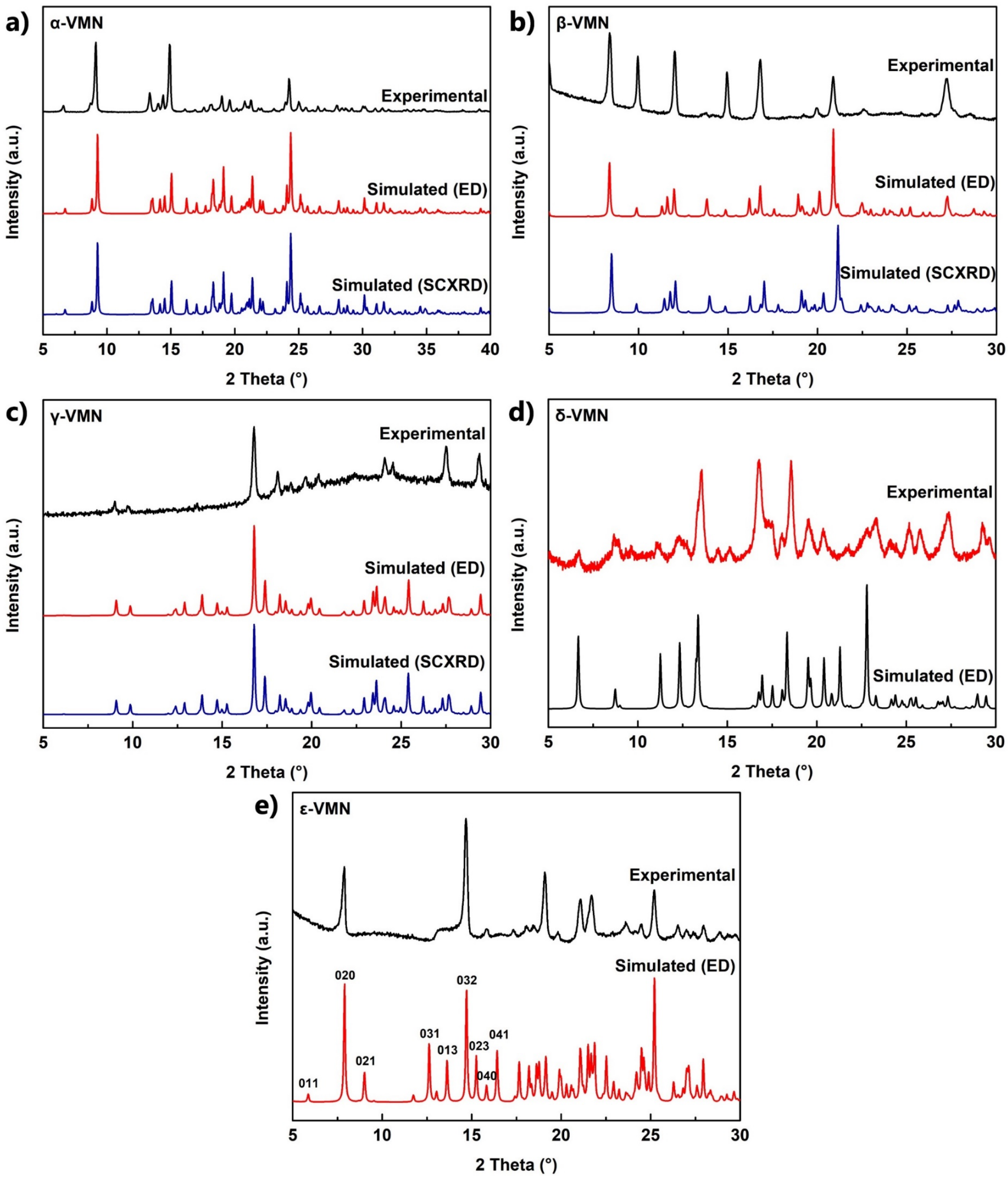

Supplementary Figure 3. Experimental and simulated powder X-ray diffraction (PXRD) patterns from the five VMN polymorph crystal structures solved using single crystal X-ray diffraction (SCXRD) and three-dimension electron diffraction (3D ED). (a) α-VMN. (b) β-VMN. (c) γ-VMN. (d) δ-VMN. (e) ε-VMN.

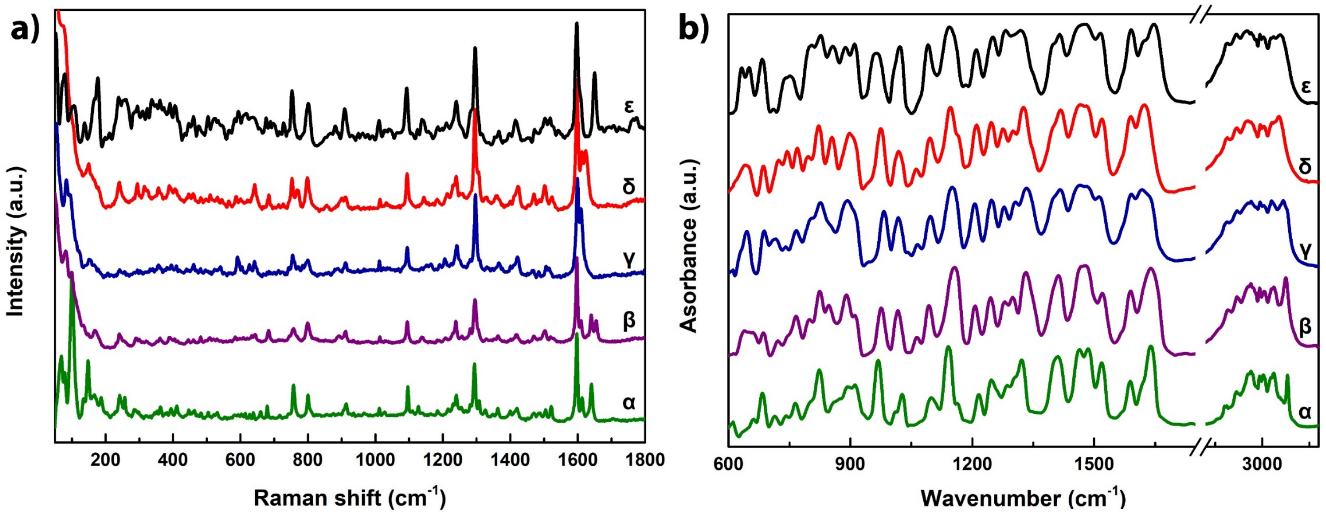

Supplementary Figure 4. Raman (a) and Fourier-transform infrared (FTIR) spectra (b) of α-, β-, γ-, δ-, and ε-VMN.

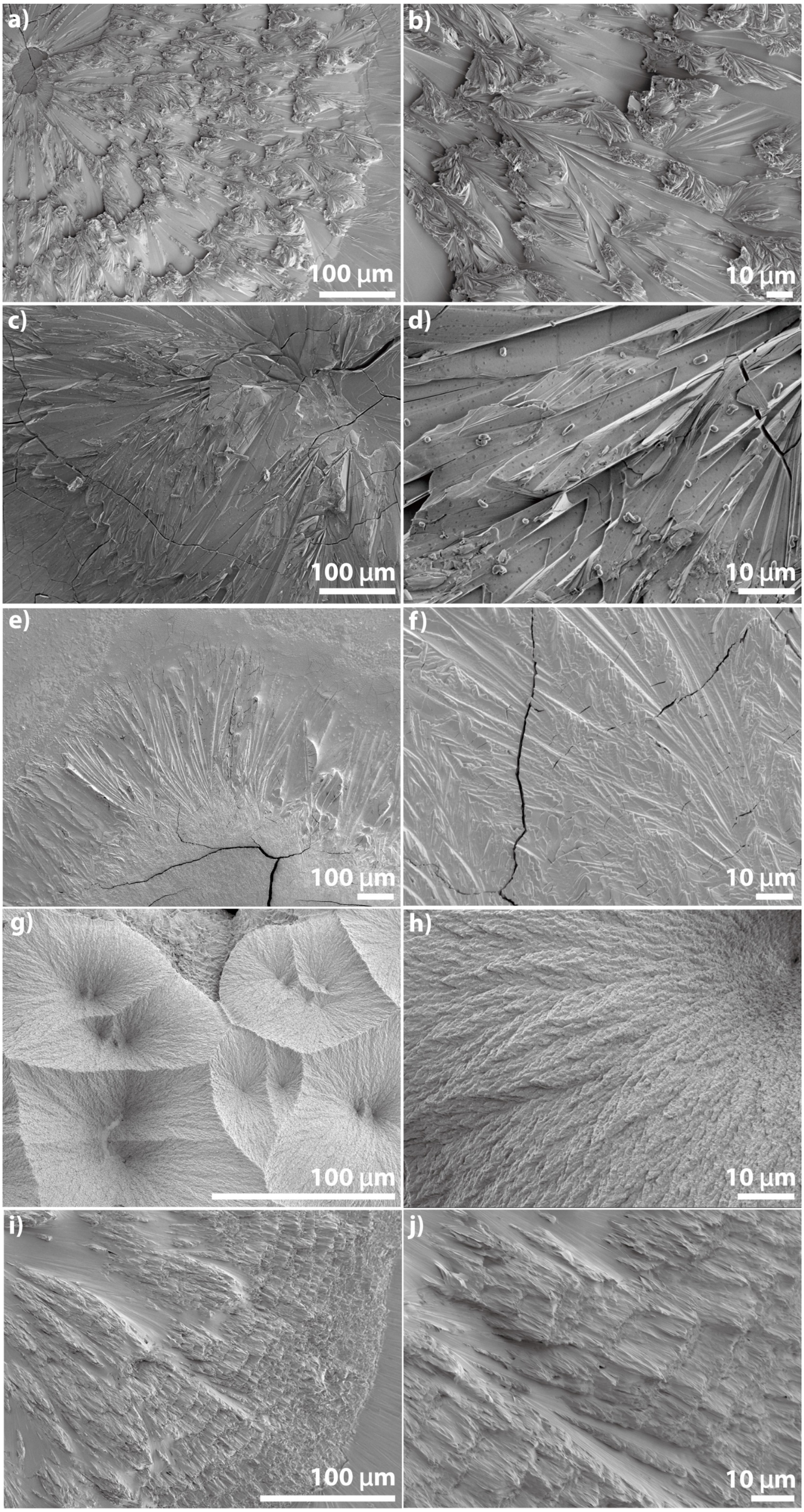

Supplementary Figure 5. Scanning electron microscopy (SEM) micrographs of VMN compact spherulites. (a-b) α-VMN. (c-d) β-VMN. (e-f) γ-VMN. (g-h) δ-VMN (i-j) ε-VMN.

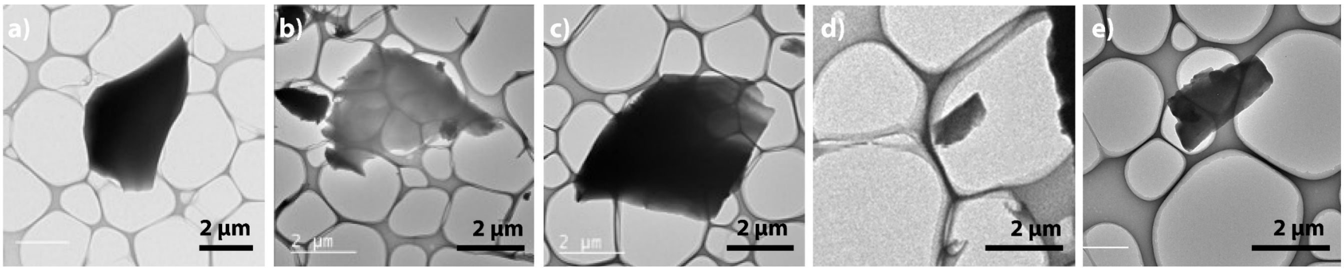

Supplementary Figure 6. Transmission electron microscopy (TEM) micrographs of the fragments of VMN compact spherulites. (a) α-VMN, (b) β-VMN, (c) γ-VMN, (d) δ-VMN, (e) ε-VMN.

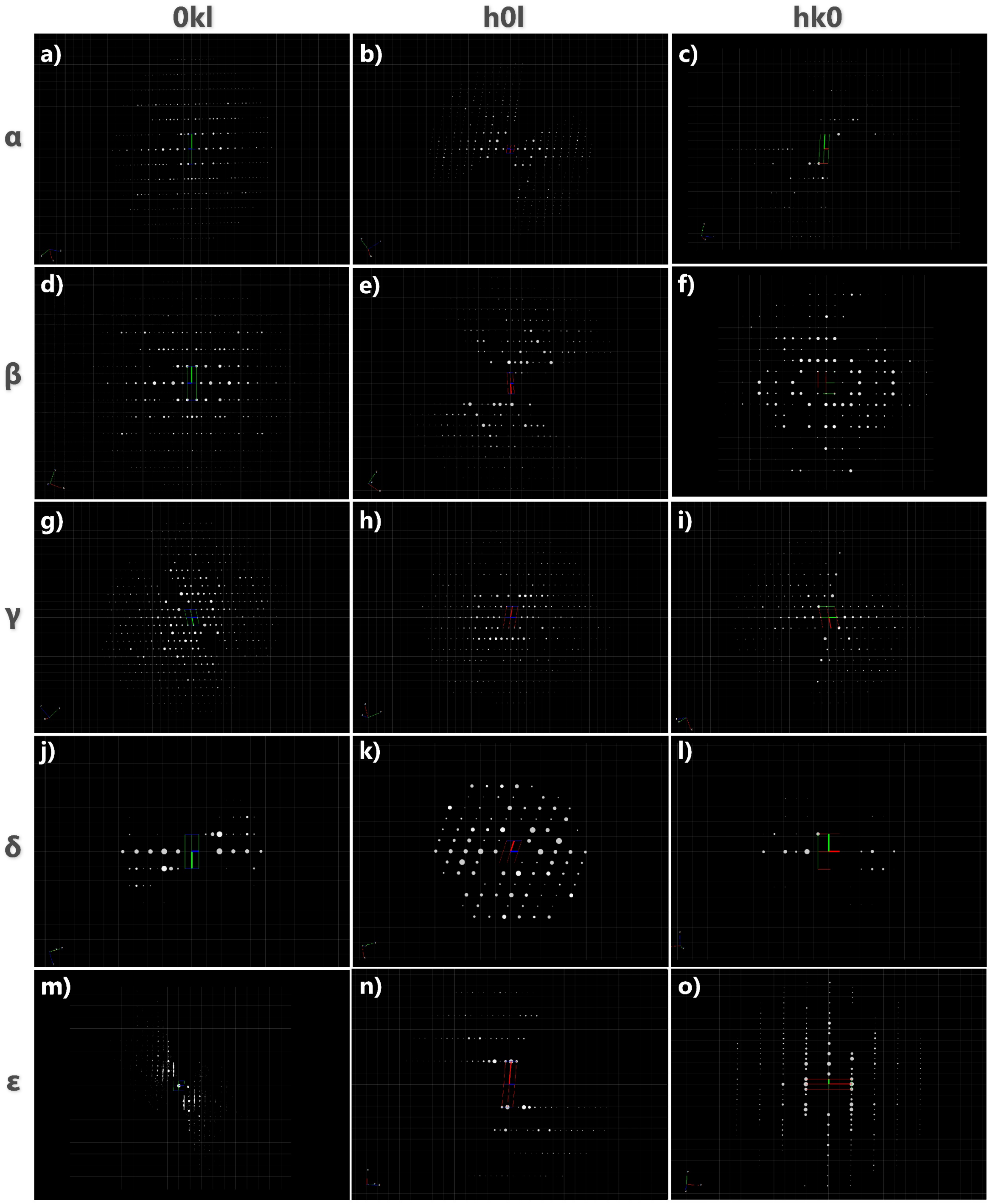

Supplementary Figure 7. 2D slices of the reciprocal lattice planes of α-, β-, γ-, δ-, and ε-VMN (compact spherulites). The unit cell parameters and space group symmetries of α-, β-, γ-VMN were taken directly from the SCXRD structures. The reflection conditions obtained from the 3D ED data of δ-VMN show that the crystal space group is $P2_1/c$ (No. 14). The reflection conditions in the 3D ED data of ε-VMN show that the crystal space group is $P2_1$ (No. 4). We note that the slices of ε-VMN were cut from different crystals.

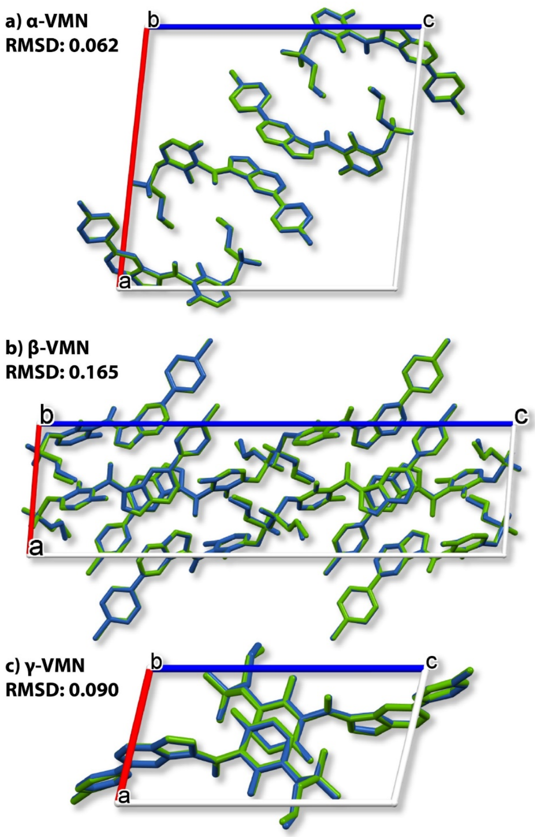

Supplementary Figure 8. Overlay of the structure models of α-, β-, and γ-VMN obtained using SCXRD (green) and 3D ED (blue).

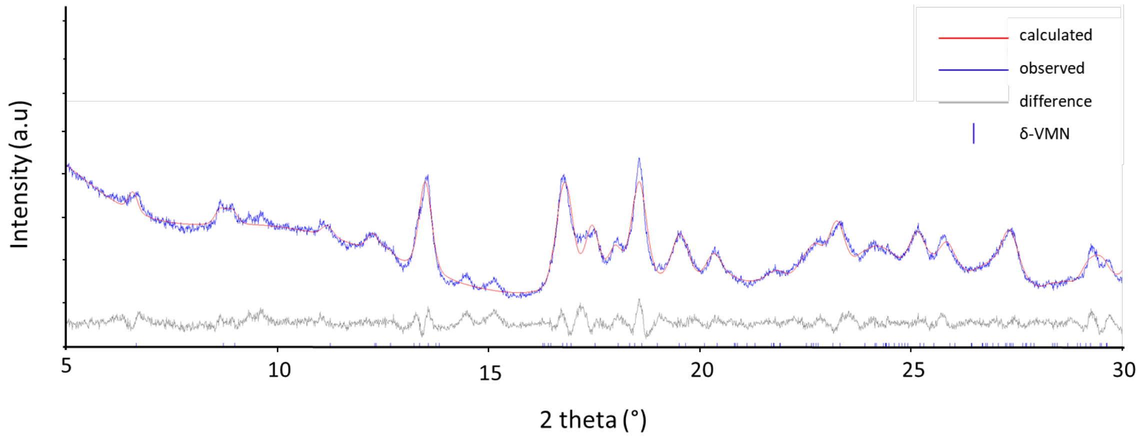

Supplementary Figure 9. Profile fit after refinement of the δ-VMN crystal structure model against the PXRD data using the Pawley method.

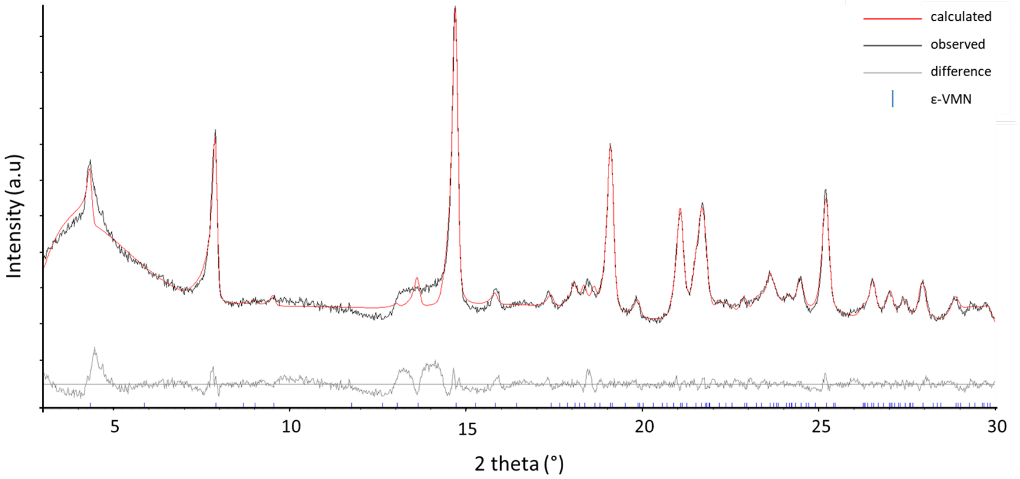

Supplementary Figure 10. Profile fit after refinement of the ε-VMN crystal structure model against the PXRD data using the Pawley method.

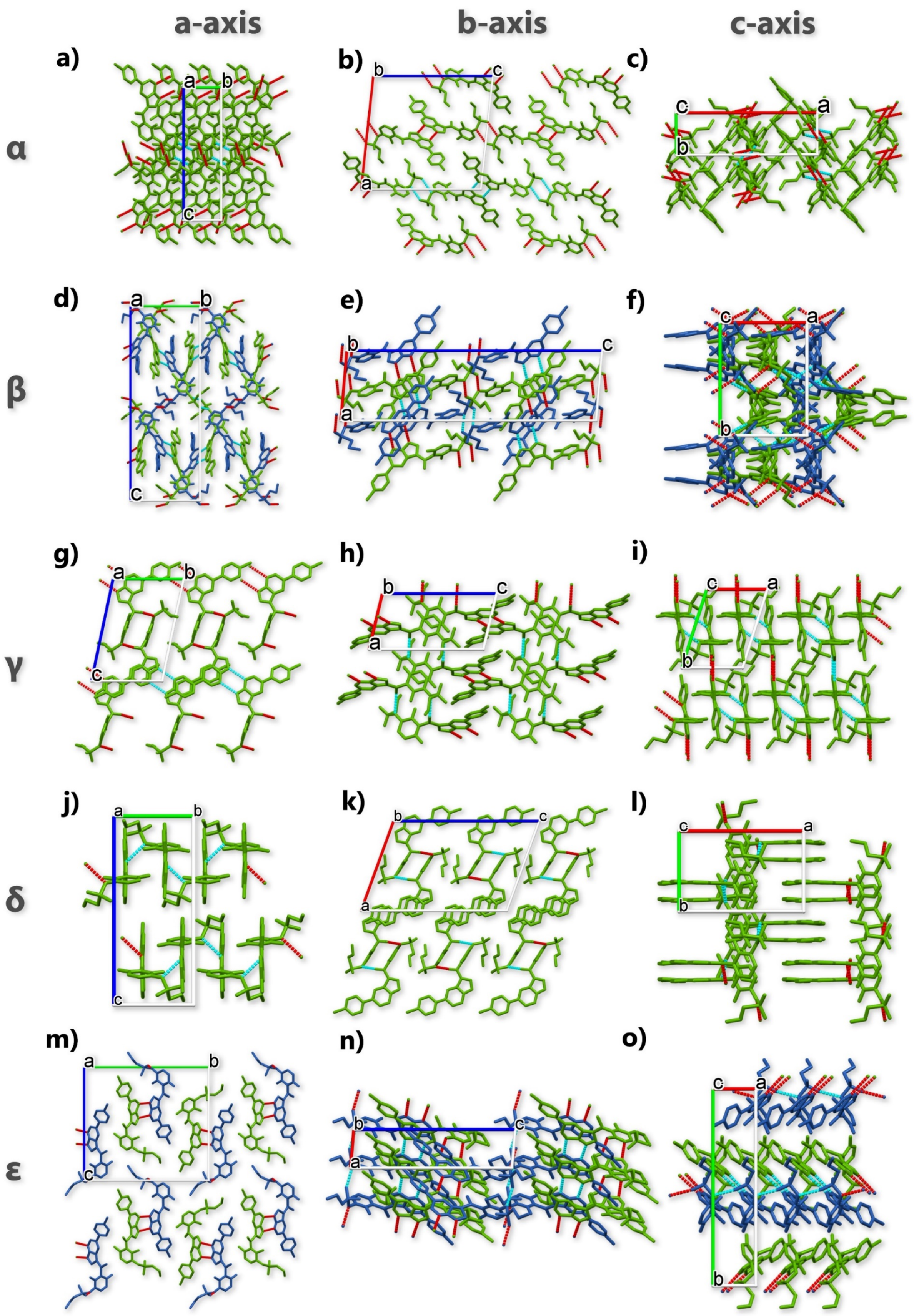

Supplementary Figure 11. Crystal structures of α-, β-, γ-, δ-, and ε-VMN viewed along the *a*-, *b*- and *c*-axes. Hydrogen bonds in red and blue indicated bonding to different molecules.

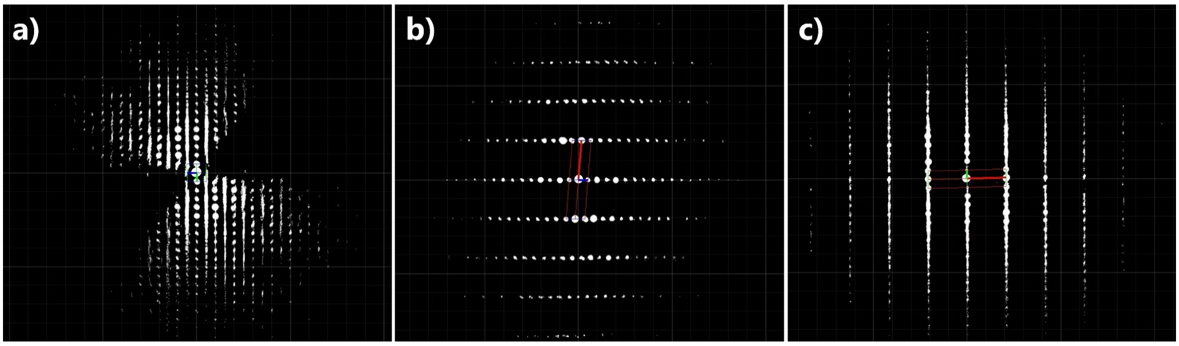

Supplementary Figure 12. 3D reciprocal lattice of ε-VMN viewed along (a) 0kl, (b) h0l, and (c) hk0, showing the diffused diffraction intensities along the *b**-axis.

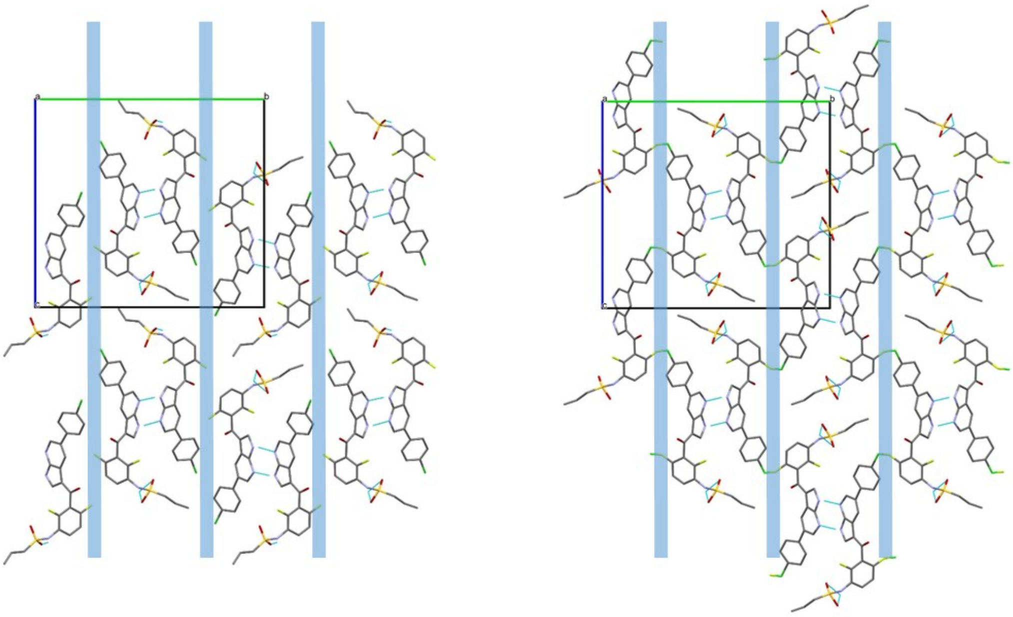

Supplementary Figure 13. The two polytypes of ε-VMM. Left: the crystal structure solved *ab initio*. Right: the proposed second polytype with a shift vector of *c* = ½. The blue lines highlight the planes of weak intermolecular interactions along the *c*-axis that allow slipping.

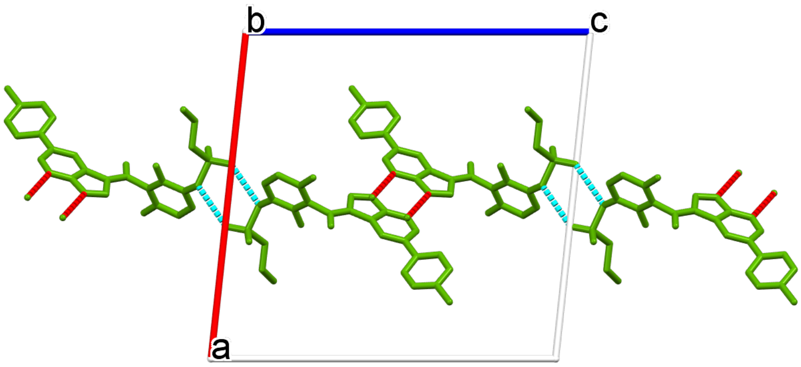

Supplementary Figure 14. Crystal packing in α-VMN.

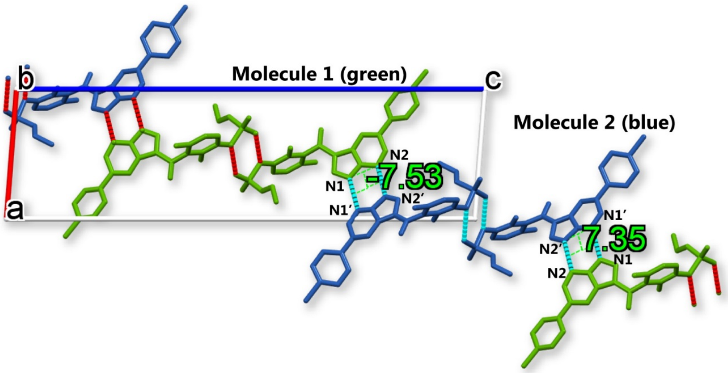

Supplementary Figure 15. Crystal packing in β-VMN with torsion angles of 7.53 ° (N2′-N2-N1-N1′) and 7.35 ° (N1-N1′-N2′-N2).

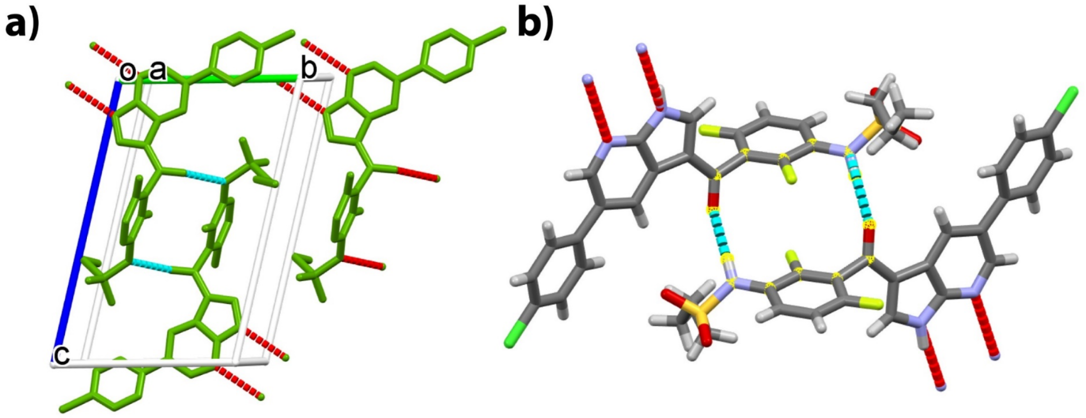

Supplementary Figure 16. Crystal packing a) and 14-membered hydrogen-bonded dimer indicated as yellow dots b) in γ-VMN.

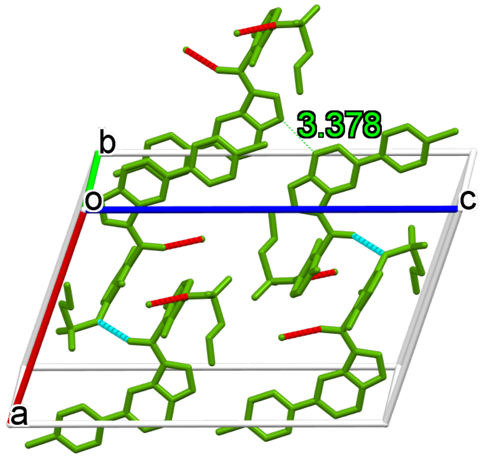

Supplementary Figure 17. Crystal packing in δ-VMN.

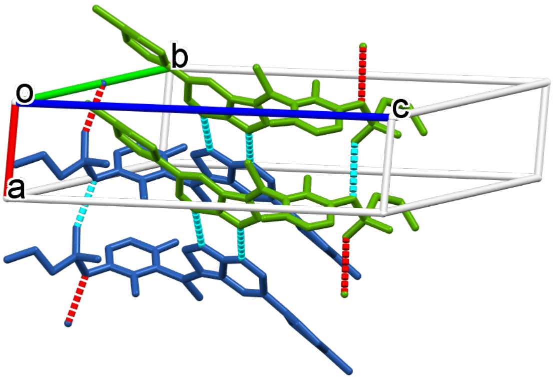

Supplementary Figure 18. Crystal packing in ε-VMN. (Molecule 1 in green and molecule 2 in blue).

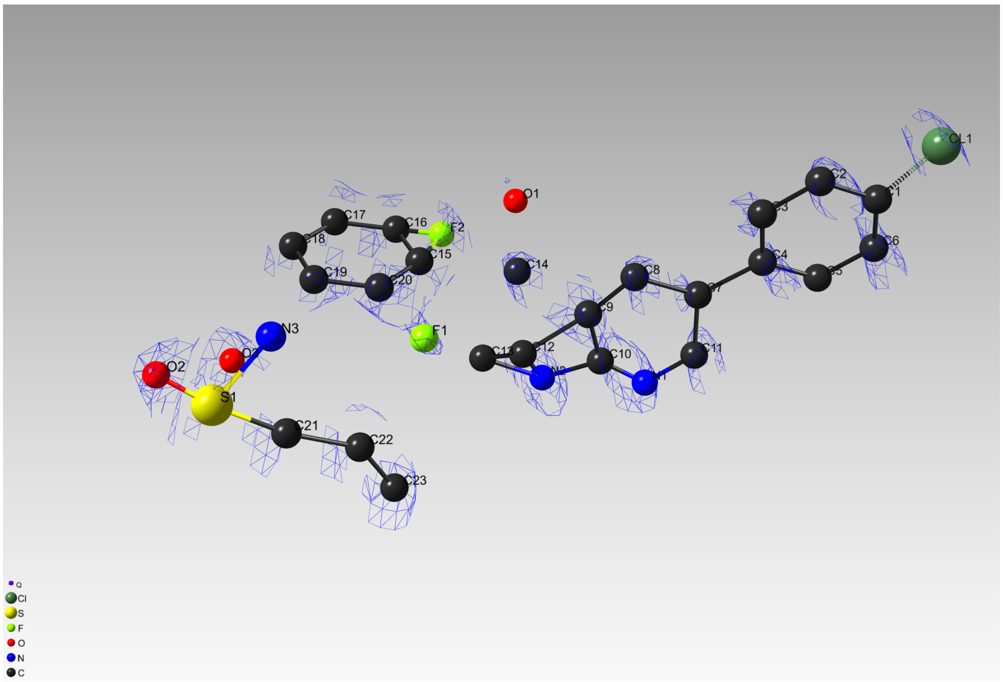

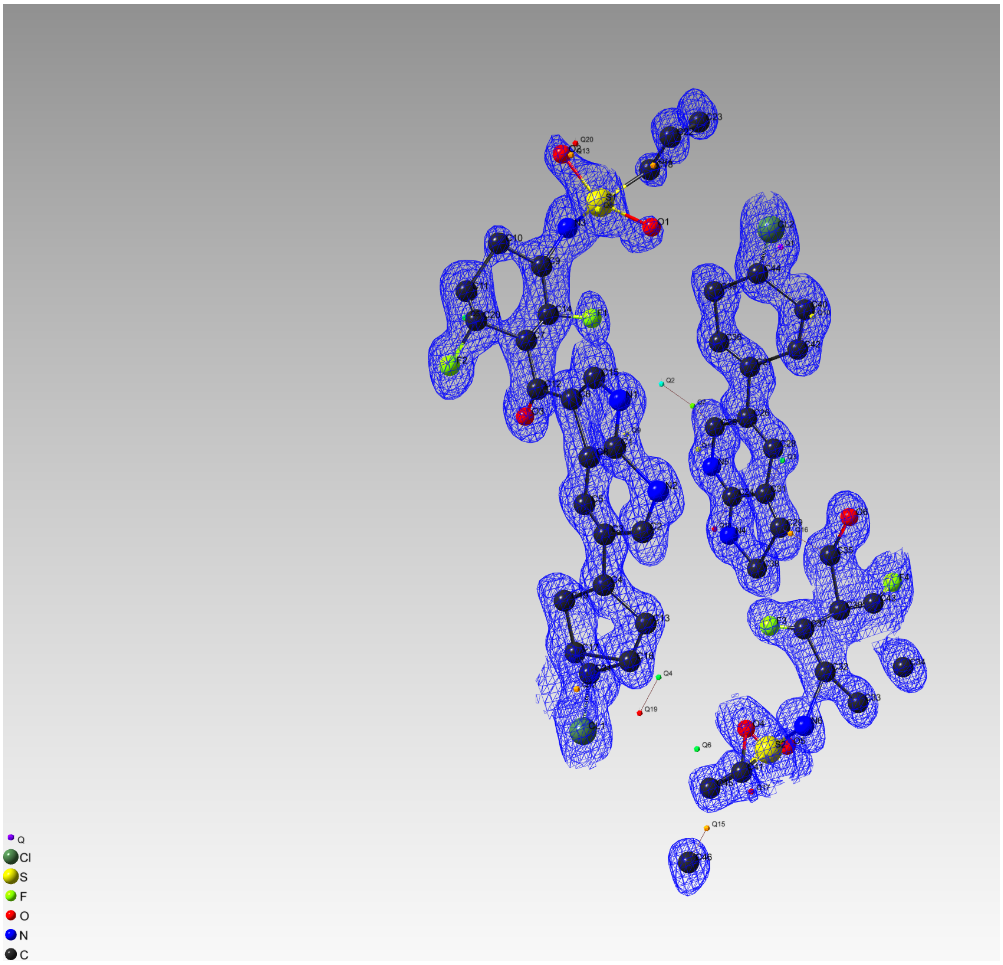

Supplementary Figure 19. Electrostatic scattering potential F(obs) map (contoured at 2σ) showing the consistency between the VMN structure models and 3D ED data. Top: δ-VMN. Down: ε-VMN.

Supplementary Table 1. Unit cell parameters of VMN polymorphs from 3D ED.

	α-VMN	β-VMN	γ-VMN	δ-VMN	ε-VMN
a (Å)	19.35(30)	10.33(10)	7.80(20)	13.99(10)	5.11(04)
b (Å)	5.86(10)	13.21(80)	10.70(30)	8.62(10)	22.95(20)
c (Å)	21.14(20)	35.82(10)	15.20(10)	21.57(10)	21.03(30)
α (°)	90	90	98.20(30)	90	90
β (°)	95.02(80)	93.93(30)	99.97(80)	109.51(90)	94.16(60)
γ (°)	90	90	104.84(80)	90	90

Supplementary Table 2. Experimental crystallographic and refinement (without restraints) statistics of δ-VMN and ε-VMN.

	δ-VMN	ε-VMN
Temperature (K)	293	293
Crystal system	Monoclinic	Monoclinic
Space group	$P2_1/c$ (14)	$P2_1$ (4)
Z', Z	1, 4	2, 4
a (Å)	14.079(1)	4.885(1)
b (Å)	8.520(1)	22.377(1)
c (Å)	21.522(1)	20.428 (1)
α (°)	90	90
β (°)	109.931(1)	94.952(2)
γ (°)	90	90
Total reflections	565	4912
Unique reflections	288	2783
Resolution (Å)	1.4	1.05
Completeness (%)	34.4	73.9
R_{int}	0.1160	0.186
I/sigma	3.66	5.30
CC1/2	98.0	97.8
No. of parameters	109	265
No. of restraints	0	1
R_1 (all)	0.2328	0.2622
No. of datasets	1	3

Supplementary Table 3. Torsion angles of VMN molecules.

	Elucidation method	ϑ1 (C12-C14-C15-C20)	ϑ2 (C3-C4-C7-C8)	ϑ3 (N3-S-C21-C22)
α-VMN	3D ED	74.97	20.20	164.60
	SCXRD	73.50	26.71	168.74
β-VMN Mol.1	3D ED	101.08	23.60	61.34
	SCXRD	100.79	22.82	61.06
β-VMN Mol.2	3D ED	60.10	34.57	147.02
	SCXRD	59.65	34.37	179.87
γ-VMN	3D ED	109.33	21.33	74.59
	SCXRD	113.99	21.85	70.96
δ-VMN	3D ED	87.38	28.62	80.75
ε-VMN Mol. 1	3D ED	78.24	21.82	160.93
ε-VMN Mol. 2	3D ED	76.16	25.35	171.75
