# Supplementary material for: Direct structure determination of vemurafenib polymorphism from compact spherulites using 3D electron diffraction
Source: Commun Chem. 2023 Jan 23;6:18. doi: 10.1038/s42004-022-00804-2 (PMC9871043; doi:10.1038/s42004-022-00804-2)
Supplement: Supplementary file 2 — Description of Additional Supplementary Files [file 42004_2022_804_MOESM2_ESM.pdf]

Description of Additional Supplementary Files

File name: Supplementary Information.pdf

Description: characterization of vemurafenib polymorphs, including POM images, PXRD patterns, Raman spectra, FTIR spectra, SEM images, TEM images and crystallographic data.

File name: Supplementary Data 1.cif

Description: experimental crystal structure of five vemurafenib polymorphs determined by SCXRD and 3D ED.

File name: Supplementary Data 2.cif

Description: energy-minimised crystal structures of five vemurafenib polymorphs by DFT method.
